# Evaluation of Pepsinogen I, II, Gastrin 17 and *Helicobacter pylori* IgG in Atrophic Gastritis: A Head‐To‐Head Comparison of Lateral Flow and Enzyme‐Linked Immunosorbent Assays

**DOI:** 10.1111/hel.70066

**Published:** 2025-08-19

**Authors:** Luochengling Xiang, Ying Zhou, Xiaopei Guo, Michiel C. Mommersteeg, Stella A. V. Nieuwenburg, Maikel P. Peppelenbosch, Manon C. W. Spaander, Gwenny M. Fuhler

**Affiliations:** ^1^ Department of Gastroenterology and Hepatology Erasmus University Medical Center Rotterdam the Netherlands

**Keywords:** atrophic gastritis, gastric cancer, gastrin, *Helicobacter pylori*, lateral flow assay, pepsinogen, surveillance

## Abstract

**Background:**

A lateral flow assay (LFA) incorporating several biomarkers, including pepsinogen I (PGI), pepsinogen II (PGII), Gastrin‐17 (G‐17), and *Helicobacter pylori* IgG, enables the rapid non‐invasive detection of atrophic gastritis (AG). However, its diagnostic performance compared to conventional enzyme‐linked immunosorbent assay (ELISA) has not been established.

**Methods:**

This head‐to‐head comparison study included participants from a prospective and multicenter cohort. Patients with gastric premalignant lesions underwent endoscopy, and fasting serum samples were collected for biomarker analysis using both LFA and ELISA.

**Results:**

A total of 204 patients were included in this study. LFA demonstrated diagnostic specificity for AG comparable to ELISA, with specificity rates of 95.74% (95% CI [85.75%–99.24%]) for LFA and 100.00% (95% CI [92.44%–100.00%]) for ELISA (*p* = 0.49). Both methods showed similar performance in detecting *H. pylori* infection, with an AUC of 0.754 (95% CI [0.616–0.891]) for LFA and 0.778 (95% CI [0.633–0.922]) for ELISA (*p* = 0.70). For identifying autoimmune gastritis in corpus AG, a reduced PGI/PGII ratio combined with elevated G‐17 levels provided excellent discrimination, achieving an AUC of 0.926 (95% CI [0.870–0.926]) for LFA and 0.924 (95% CI [0.861–0.924]) for ELISA.

**Conclusion:**

The LFA assay is a feasible, rapid, and non‐invasive tool for assessing gastric functional mucosa. Its diagnostic performance for detecting AG is comparable to ELISA, making it a supplementary tool in point‐of‐care settings to improve the early detection of AG.

**Trial Registration:**

This study was not registered as a clinical trial, as it is based on an observational study, Progression and Regression of precancerous Gastric Lesions (PROREGAL) study.

## 1 Introduction

Gastric cancer is one of the most prevalent malignancies and ranks as the fourth leading cause of cancer‐related mortality worldwide [1]. Chronic *Helicobacter pylori* ( *H. pylori* ) infection is a well‐established causal factor of gastric carcinogenesis. Persistent infection can initiate a multistep process, known as the Correa cascade, that progresses from atrophic gastritis (AG) to intestinal metaplasia (IM), dysplasia, and eventually gastric cancer [2]. Early detection of these premalignant lesions is critical for effective intervention, yet many individuals remain asymptomatic and undiagnosed until the disease reaches an advanced stage.

AG represents the initial step in the multistep neoplastic progression, characterized by the loss of normal gastric glands, which are replaced by connective tissues or non‐native epithelium. The primary causes of AG are chronic *H. pylori* infection and autoimmunity. *H. pylori* ‐associated AG typically originates in the incisura and antrocorporal transition mucosa, gradually spreading to the corpus along the lesser curvature over time. In contrast, autoimmune gastritis (AIG) is less common and is characterized by corpus‐predominant atrophy, driven by lymphocyte‐mediated destruction of oxyntic glands due to autoimmunity against acid‐secreting parietal cells [3].

Non‐invasive tests have emerged as valuable tools for identifying high‐risk patients and guiding endoscopic surveillance, thereby enhancing the early detection of gastric cancer [4]. Among these, serum pepsinogens are well‐established biomarkers for severe AG [5]. Pepsinogen I (PGI), secreted exclusively by corpus chief cells, and pepsinogen II (PGII), produced throughout the gastric mucosa and duodenum, serve as key indicators. A low serum PGI level and/or a reduced PGI/PGII ratio suggest advanced AG. In such cases, the European management of epithelial precancerous conditions and lesions in the stomach (MAPS) III Guideline recommends endoscopy evaluation, regardless of *H. pylori* infection status [6]. Gastrin‐17 (G‐17), secreted by antral G cells, reflects the functional status of the antral mucosa. Low serum G‐17 levels may result from hyperchlorhydria‐induced suppression or antral atrophy. G‐17 is often combined with pepsinogens to provide a comprehensive assessment of both antral and corpus mucosal integrity [7].

ELISA‐based evaluation of these serum markers has shown promise in identifying AG and stratifying high‐risk patients [8, 9, 10]. However, these assays require laboratory infrastructure and skilled personnel, which limits their accessibility in resource‐limited settings [11]. Lateral flow assays (LFA) provide a simpler and faster alternative that could be performed outside traditional laboratory environments [12]. LFA gained widespread attention during the COVID‐19 pandemic, demonstrating their effectiveness for large‐scale testing. This success has spurred interest in expanding their use to other diagnostic applications [13]. Recently, an LFA‐based assessment for AG has been developed, incorporating PGI, PGII, PGI/PGII ratio, G‐17, and *H. pylori* IgG as biomarkers. However, its diagnostic performance compared to conventional ELISA remains to be evaluated.

Therefore, this study aimed to directly assess the diagnostic performance of LFA in detecting AG and to compare its performance with ELISA, establishing a basis for its potential clinical application.

## 2 Methods

### 2.1 Study Population

This study was based on the ongoing prospective Progression and Regression of precancerous Gastric Lesions (PROREGAL) study, initiated in 2009 across six hospitals (one academic and five regional) in the Netherlands and one regional hospital in Norway. Detailed descriptions of the study design have been published previously [14, 15]. It includes patients over 18 years old with a prior endoscopy diagnosis of gastric premalignant lesions (atrophic gastritis, intestinal metaplasia, or dysplasia). Exclusion criteria include prior upper gastrointestinal surgery, gastric carcinoma or any other malignancy not being in remission, severe comorbidity limiting the expected survival to less than two years, portal hypertension, or a *CDH1* mutation.

According to the study protocol, surveillance endoscopies were performed following the index endoscopy, adhering to the standardized biopsy protocol for the histological evaluation. Random biopsies were taken from five standardized intragastric locations (the antrum, the angulus, the corpus grater curvature, the corpus lesser curvature and the cardia), and targeted biopsies were taken from endoscopically visible abnormalities before random biopsies collection. All biopsies were analyzed for *H. pylori* colonization by pathology. The histological assessment followed the updated Sydney classifications, with atrophic gastritis severity scored using the operative link on gastritis assessment (OLGA) and IM assessed through the operative link on gastric intestinal metaplasia (OLGIM) systems [16, 17].

Fasting serum samples were collected on the day of endoscopy and stored at −80°C for subsequent analysis. Written informed consent was obtained, and the study was approved by the Medical Ethical Review Committee of Erasmus MC (MEC‐2009‐090).

### 2.2 Serological Measurement

Serum samples were analyzed using both lateral flow assay (LFA, Biohit GastroPanel quick test NT, Biohit, Helsinki, Finland) and enzyme‐linked immunosorbent assay (GastroPanel ELISA, Biohit, Helsinki, Finland). Both tests were performed on the same freeze–thaw cycle; all procedures were implemented according to the manufacturer's instructions.

For LFA, diluted serum sample was added to the sample windows in the test cassette. This cassette was incubated in a dark environment for 15 min, and fluorescent signals were quantified by the GP reader NT (Biohit, Helsinki, Finland). For ELISA, the serum sample was diluted at 1:5 for G‐17, 1:20 for PGI and PGII, and 1:400 for *H. pylori* IgG. Each control and diluted serum sample was measured in duplicate according to the manufacturer's protocol. Absorbance was measured by a spectrophotometer (Infinite 200 Pro plate reader, Tecan Group Ltd.) at a wavelength of 450 nm.

The manufacturer‐recommended cutoff values were applied: PGI < 30 μg/L, PGII < 3 μg/L, PGI/PGII ratio < 3, G‐17 < 1.8 ρmol/L for LFA and G‐17 < 1 ρmol/L for ELISA. *H. pylori* IgG titers above 25 EIU for LFA or 30 EIU for ELISA were considered positive for *H. pylori* infection. The GastroPanel algorithm was followed according to the manufacturer's instructions to distinguish corpus‐restricted, antrum‐restricted, and AG in the antrum and corpus from non‐atrophy.

### 2.3 Statistical Analysis

Continuous variables were presented as the median with the interquartile range (IQR). Normality of data was assessed using Shapiro‐Wilkes tests. Pairwise Spearman rank correlations were calculated to assess the relationships between individual markers measured by LFA and ELISA. Categorical variables were compared using chi‐square tests, Fisher's exact tests, or McNemar's tests as appropriate. The agreement between LFA and ELISA was assessed by Cohen's kappa coefficient. Sensitivity, specificity, and accuracy were calculated using histology as the gold standard. Positive predictive value (PPV), negative predictive value (NPV), and the receiver operating characteristic (ROC) curves with corresponding areas under the curve (AUC) were used to evaluate the diagnostic performance. DeLong's tests compared differences between ROC curves, and Youden's index determined the best cutoff values. Two‐sided *p‐*values < 0.05 were considered significant, and all analyses were performed using GraphPad Prism 10.1.2 and R version 4.4.1.

## 3 Results

### 3.1 Patient Characteristics

A total of 204 patients (50% males, median age 65.0 ± 14.5) without dysplasia or gastric cancer were included in this study. The baseline characteristics are summarized in Table 1. Histopathological analysis identified 157 patients (77.0%) with AG, including 51 patients (25.0%) with antrum‐restricted AG, 9.8% with corpus‐restricted AG, and 42.2% with AG in both antrum and corpus. In this study, the incisura was not classified as part of either the antrum or the corpus. The OLGA and OLGIM staging systems were applied to assess the severity of gastritis. The OLGA system classified 115 (56.4%) patients as stage 0, 23 (11.3%) as stage I, 14 (6.9%) as stage II, 6 (2.9%) as stage III, and 2 (1.0%) as stage IV. OLGIM staging classified 84 (41.2%) patients as stage 0, 39 (19.1%) as stage I, 48 (23.5%) as stage II, 26 (12.7%) as stage III, and 5 (2.5%) as stage IV.

**TABLE 1:** Baseline characteristics of the study population.

	PROREGAL cohort (*n* = 204)
Gender, *n* (%)	
Male	101 (49.5)
Female	103 (50.5)
Median age, years (IQR)	65.0 (14.5)
Ethnicity (%)	
Caucasian	179 (87.7)
Non‐Caucasian	23 (11.3)
Missing	2 (1.0)
Atrophy (%)	
Antrum	51 (25.0)
Corpus	20 (9.8)
Antrum and corpus	86 (42.2)
OLGA classification (%)	
0	115 (56.4)
I	23 (11.3)
II	14 (6.9)
III	6 (2.9)
IV	2 (1.0)
Missing	44 (21.6)
OLGIM classification (%)	
0	84 (41.2)
I	39 (19.1)
II	48 (23.5)
III	26 (12.7)
IV	5 (2.5)
Missing	2 (1.0)

Abbreviations: IQR, interquartile range; OLGA, operative link on gastritis assessment; OLGIM, operative link on gastric intestinal metaplasia.

### 3.2 LFA and ELISA Provide Comparable Diagnostic Information for Atrophic Gastritis

**FIGURE 1:**
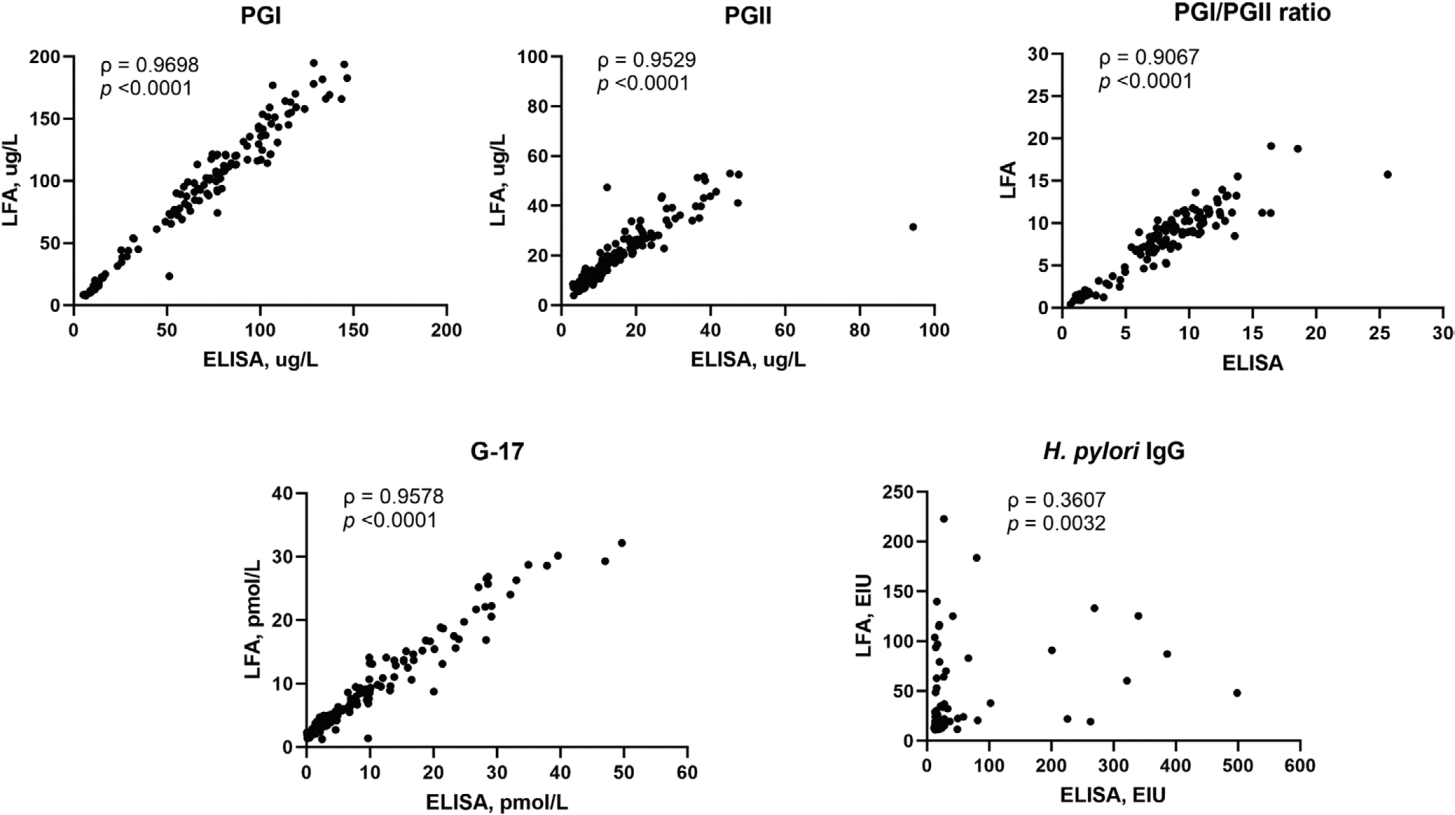
Pairwise Spearman correlation between LFA and ELISA measurements for pepsinogen I (PGI), pepsinogen II (PGII), PGI/PGII ratio, gastrin‐17 (G‐17), and *Helicobacter pylori* ( *H. pylori* ) IgG between LFA and ELISA. Strong correlations were observed for PGI (ρ = 0.9698), PGII (ρ = 0.9529), PGI/PGII ratio (ρ = 0.9067), G‐17 (ρ = 0.9578), while *H. pylori* IgG showed a weak correlation (ρ = 0.3607).

To evaluate the diagnostic performance of LFA compared to ELISA, serum samples were analyzed for PGI, PGII, PGI/PGII ratio, G‐17, and *H. pylori* IgG. After excluding samples above the limit of detection, the pair‐wise correlation analysis included 123 pairs for PGI, 197 pairs for PGII, 123 pairs for PGI/PGII ratio, 131 pairs for G‐17, and 65 pairs for *H. pylori* IgG. Scatter plots (Figure 1) demonstrated strong correlations for most markers, with Spearman rank correlation coefficients of 0.9698 for PGI, 0.9529 for PGII, 0.9067 for PGI/PGII ratio, and 0.9578 for G‐17. However, *H. pylori* IgG exhibited a weaker correlation, with a Spearman's rank correlation coefficient of 0.3607, indicating a moderate monotonic association between the two tests.

**FIGURE 2:**
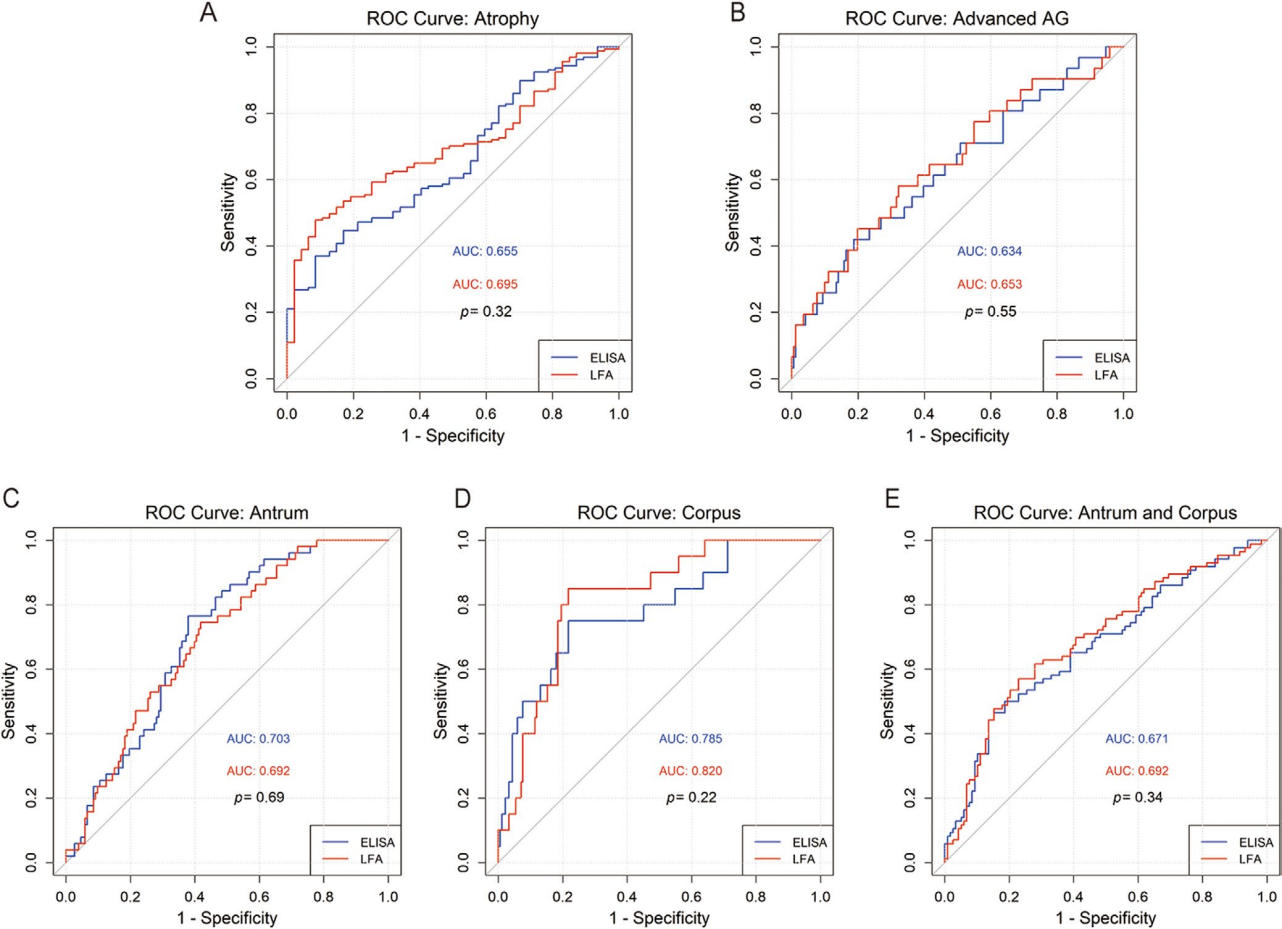
Receiver operating characteristic (ROC) curves comparing the diagnostic performance of LFA and ELISA in detecting atrophic gastritis (AG). (A, B) ROC curves for LFA and ELISA in detecting overall AG, with area under curve (AUC) of 0.695 and 0.655, respectively, and advanced AG, with AUC of 0.653 and 0.634. (C‐E) ROC curves for LFA and ELISA in detecting location‐specific AG, with AUC values of 0.692 and 0.703 for antrum‐restricted AG (C), 0.820 and 0.785 for corpus‐restricted AG (D), and 0.692 and 0.671 for AG affecting both the antrum and corpus (E).

Using the GastroPanel algorithm (Figure S1), AG was classified into four categories: non‐atrophy, antrum‐restricted AG, corpus‐restricted AG, and AG in both antrum and corpus. Table 2 summarizes the diagnostic performance of LFA and ELISA for AG detection. Both assays demonstrated similarly high specificity, with LFA at 95.74% (95% CI [85.75%–99.24%]) and ELISA at 100.00% (95% CI [92.44%–100.00%]) (*p* = 0.49). Sensitivity was also comparable between LFA (23.57%, 95% CI [17.61%–30.79%]) and ELISA (27.39%, 95% CI [21.02%–34.84%]) (*p* = 0.44). Overall diagnostic accuracy showed no significant differences between LFA (40.20%, 95% CI [33.41%–47.27%]) and ELISA (44.12%, 95% CI [37.19%–51.22%]) (*p* = 0.45). ROC curves for AG diagnosis (Figure 2A) indicated comparable AUC for LFA (0.695, 95% CI [0.619–0.695]) and ELISA (0.655, 95% CI [0.572–0.655]) (*p* = 0.32).

**TABLE 2:** Diagnostic performance of GastroPanel LFA and ELISA for the detection of atrophic gastritis.

	LFA (%) (95% CI)	ELISA (%) (95% CI)	*p*
AG			
Sensitivity	23.57 (17.61–30.79)	27.39 (21.02–34.84)	0.44
Specificity	95.74 (85.75–99.24)	100.00 (92.44–100.00)	0.49
PPV	94.87 (83.11–99.09)	100.00 (91.80–100.00)	0.22
NPV	27.27 (21.05–34.53)	29.19 (22.72–36.63)	0.70
Accuracy	40.20 (33.41–47.27)	44.12 (37.19–51.22)	0.45
Advanced AG			
Sensitivity	32.26 (18.57–49.86)	35.48 (21.12–53.05)	0.79
Specificity	95.74 (85.75–99.24)	100.00 (92.44–100.00)	0.49
PPV	83.33 (55.20–97.04)	100.00 (74.12–100.00)	0.48
NPV	68.18 (56.21–78.15)	70.15 (58.34–79.77)	0.81
Accuracy	70.51 (59.11–80.30)	74.36 (63.21–83.58)	1.00

Abbreviations: AG, atrophic gastritis; CI, confidence interval; ELISA, enzyme‐linked immunosorbent assay; LFA, lateral flow assay; NPV, negative predictive value; PPV, positive predictive value.

The OLGA and OLGIM staging systems were applied to evaluate the severity of mucosal changes. Among the study population, 8 patients (3.9%) were classified as OLGA III/IV, and 31 patients (15.2%) were classified as OLGIM III/IV. Due to the small number of OLGA III/IV cases and MAPS III Guideline recommending OLGIM over OLGA for mucosal changes [6], OLGIM III/IV cases were considered indicative of advanced AG. Both LFA (70.51%, 95% CI [59.11%–80.30%]) and ELISA (74.36%, 95% CI [63.21%–83.58%]) showed higher accuracy in detecting advanced AG compared to overall AG. However, the difference was not statistically significant (*p* = 0.44 for LFA and *p* = 0.51 for ELISA). Similarly, ROC curves (Figure 2B) showed comparable performance for advanced AG, with AUC of 0.653 (95% CI [0.542–0.653]) for LFA (*p* = 0.54) and 0.634 (95% CI [0.521–0.634]) for ELISA (*p* = 0.77).

The diagnostic performance of LFA and ELISA was further investigated for the location‐specific AG, including antrum‐restricted AG, corpus‐restricted AG, and AG in the antrum and corpus. The results are summarized in Table 3. No significant differences in performance were observed between the two methods. Both assays demonstrated high specificity across all categories, though sensitivity varied. LFA (*p* < 0.01) and ELISA (*p* < 0.01) demonstrated higher sensitivity for corpus‐restricted AG (40.00%, 95% CI [21.88%–61.34%] for both) compared to antrum‐restricted AG (3.92%, 95% CI [0.70%–13.22%] for LFA; 1.96%, 95% CI [0.10%–10.30%], for ELISA). Lower sensitivity for antrum‐restricted AG likely contributed to poor sensitivity for AG in the antrum and corpus (3.49%, 95% CI [0.95%–9.76%] for LFA; 4.65%, 95% CI [1.82%–11.36%] for ELISA).

**TABLE 3:** Diagnostic performance of GastroPanel LFA and ELISA for the location‐specific atrophic gastritis.

	LFA (%) (95% CI)	ELISA (%) (95% CI)	*p*
Antrum‐restricted AG			
Sensitivity	3.92 (0.70–13.22)	1.96 (0.10–10.30)	1.00
Specificity	97.39 (93.47–98.98)	96.08 (91.71–98.19)	0.88
PPV	33.33 (5.92–70.00)	14.29 (0.73–51.31)	0.56
NPV	75.25 (68.80–80.74)	74.62 (68.11–80.19)	0.88
Accuracy	74.02 (67.43–79.89)	72.55 (65.88–78.55)	0.45
Corpus‐restricted AG			
Sensitivity	40.00 (21.88–61.34)	40.00 (21.88–61.34)	1.00
Specificity	89.13 (83.81–92.85)	88.59 (83.18–92.41)	0.99
PPV	28.57 (15.25–47.06)	27.59 (14.70–45.72)	0.93
NPV	93.18 (88.46–96.06)	93.14 (88.40–96.03)	0.99
Accuracy	84.31 (78.58–89.02)	83.82 (78.04–88.60)	0.45
Atrophy in antrum and corpus			
Sensitivity	3.49 (0.95–9.76)	4.65 (1.82–11.36)	1.00
Specificity	98.31 (94.03–99.70)	97.46 (92.79–99.31)	0.99
PPV	60.00 (23.07–92.89)	57.14 (25.05–84.18)	1.00
NPV	58.29 (51.35–64.92)	58.38 (51.40–65.03)	0.99
Accuracy	58.33 (51.24–65.18)	58.33 (51.24–65.18)	0.45

Abbreviations: AG, atrophic gastritis; CI, confidence interval; ELISA, enzyme‐linked immunosorbent assay; LFA, lateral flow assay; NPV, negative predictive value; PPV, positive predictive value.

ROC curve analysis revealed that LFA demonstrated superior discrimination for corpus‐restricted AG (AUC = 0.820, 95% CI [0.734–0.820]) compared to antrum‐restricted AG (AUC = 0.692, 95% CI [0.615–0.692], *p* = 0.03) and AG in both antrum and corpus (AUC = 0.692, 95% CI [0.618–0.692], *p* = 0.03) (Figure 2C–E). Similarly, ELISA showed improved discrimination for corpus‐restricted AG (AUC = 0.785, 95% CI [0.671–0.785]) relative to antrum‐restricted AG (AUC = 0.703, 95% CI [0.629–0.703], *p* = 0.24) and AG in the antrum and corpus (AUC = 0.671, 95% CI [0.595–0.671], *p* = 0.10), although these differences did not reach statistical significance.

### 3.3 Etiological Factors in Atrophic Gastritis May Be Determined Using LFA and ELISA

**FIGURE 3:**
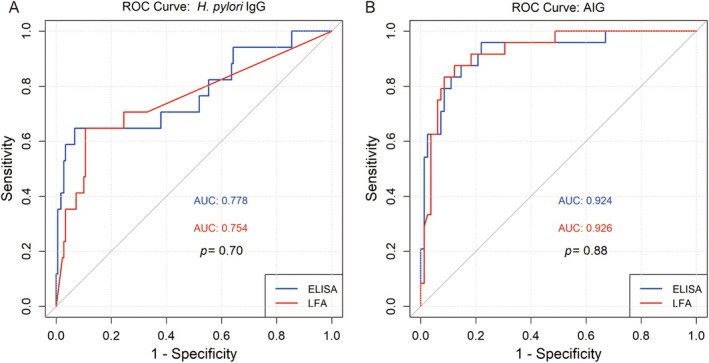
Etiological factors determined by LFA and ELISA. (A) ROC curves of LFA and ELISA demonstrated comparable discrimination in *Helicobacter pylori* ( *H. pylori* ) infection, with area under curve (AUC) of 0.754 and 0.778, respectively. (B) PGI/PGII ratio and G‐17 measured by LFA and ELISA provided similar excellent discrimination between autoimmune gastritis (AIG) and non‐autoimmune gastritis (non‐AIG), with AUC of 0.926 and 0.924, respectively.

To explore the etiological applications of LFA and ELISA in atrophic gastritis, we compared their diagnostic performance of *H. pylori* IgG, using histological *H. pylori* positivity as the gold standard. Given the weaker correlation between LFA and ELISA, Cohen's kappa coefficient was calculated to evaluate the agreement in qualitative outcomes based on the recommended cutoff values. The agreement for *H. pylori* IgG was fair (к = 0.393, 95% CI [0.231–0.555]). Despite this discrepancy, ROC analysis (Figure 3A) revealed comparable discriminatory performance, with AUC of 0.754 (95% CI [0.616–0.891]) for LFA and 0.778 (95% CI [0.633–0.922]) for ELISA (*p* = 0.70).

To enhance diagnostic performance, the best cutoff values for *H. pylori* IgG were determined by Youden's index. Adjusting the LFA cutoff from 25.0 EIU to 37.4 EIU improved specificity from 85.47% (95% CI [79.57%–89.89%]) to 89.39% (95% CI [84.02%–93.10%]) (*p* < 0.05) and accuracy from 83.67% (95% CI [77.74%–88.56%]) to 87.24% (95% CI [81.75%–91.57%]) (*p* < 0.05). Similarly, increasing the ELISA cutoff to 60.0 EIU enhanced specificity from 86.59% (95% CI [80.83%–90.82%]) to 94.97% (95% CI [90.72%–97.33%]) (*p* < 0.01) and accuracy from 84.69% (95% CI [78.88%–89.43%]) to 91.84% (95% CI [87.08%–95.26%]) (*p* < 0.01) (Table 4).

**TABLE 4:** Diagnostic performance of LFA and ELISA for *H. pylori* infection.

	LFA			ELISA		
	Recommended (≥ 25 EIU)	Best cutoff (≥ 37.4 EIU)	*p*	Recommended (≥ 30 EIU)	Best cutoff (≥ 60.0 EIU)	*p*
Sensitivity (%) (95% CI)	64.71 (41.30–82.69)	64.71 (41.30–82.69)	—	64.71 (41.30–82.69)	58.82 (36.01–78.39)	—
Specificity (%) (95% CI)	85.47 (79.57–89.89)	89.39 (84.02–93.10)	< 0.05	86.59 (80.83–90.82)	94.97 (90.72–97.33)	< 0.01
PPV (%) (95% CI)	29.73 (17.49–45.78)	36.67 (21.87–54.49)	—	31.43 (18.55–47.98)	52.63 (31.71–72.67)	—
NPV (%) (95% CI)	96.23 (92.01–98.26)	96.39 (92.34–98.33)	—	96.27 (92.11–98.28)	96.05 (92.06–98.07)	—
Accuracy (%) (95% CI)	83.67 (77.74–88.56)	87.24 (81.75–91.57)	< 0.05	84.69 (78.88–89.43)	91.84 (87.08–95.26)	< 0.01

Abbreviations: CI, confidence interval; ELISA, enzyme‐linked immunosorbent assay; *H. pylori, Helicobacter pylori*; LFA, lateral flow assay; NPV, negative predictive value; PPV, positive predictive value.

Autoimmune gastritis, characterized by the immune‐mediated destruction of parietal cells in the oxyntic mucosa, is another key etiological factor for AG, often resulting in corpus‐predominant atrophy. MAPS III Guideline recommends endoscopic surveillance every 3 years for individuals with AIG to monitor disease progression [6]. In this cohort, AIG patients were identified by anti‐parietal cell antibodies (APCA) enzyme fluoroimmunoassay [18]. Given the corpus‐predominant nature of AIG, cases of corpus‐restricted AG and AG involving both the antrum and corpus were included as corpus AG for subsequent analysis. Among these, 24 AIG patients were compared with non‐autoimmune gastritis to evaluate the biomarkers' predictive performance.

Multivariant logistic regression identified PGI/PGII ratio and G‐17 as independent predictors of AIG. A lower PGI/PGII ratio (*p* = 0.03 for LFA and ELISA) and elevated G‐17 levels (*p* < 0.01 for LFA and ELISA) were significantly associated with AIG. These predictors were combined to generate ROC curves, showing excellent discrimination for AIG in corpus AG, with AUC of 0.926, 95% CI [0.870–0.926] for LFA and 0.924, 95% CI [0.861–0.924] for ELISA (Figure 3B). DeLong's test indicated no significant difference between the methods (*p* = 0.88), suggesting that LFA and ELISA provide comparable Supporting Information in detecting AIG.

## 4 Discussion

In this study, we evaluated the diagnostic performance of LFA as a novel, rapid, and non‐invasive test for AG, directly comparing it to the established ELISA. To our knowledge, this is the first head‐to‐head comparison of these two diagnostic approaches in individuals with histologically confirmed AG. Our findings demonstrate that LFA offers comparable accuracy and specificity to ELISA in detecting AG. Notably, our analysis confirmed that combining PGI/PGII ratio and G‐17 in LFA maintains its efficacy in detecting AIG, consistent with previous findings for ELISA tests [18]. These results suggest that LFA could serve as a valuable alternative to ELISA, particularly in settings where rapid and point‐of‐care testing is essential.

Previous research has shown that the diagnostic accuracy of the GastroPanel algorithm varies with the location of the gastric atrophy, with the strongest performance in identifying severe AG and corpus atrophy [8, 19]. Our findings align with these reports, demonstrating that LFA performs as effectively as ELISA in diagnosing AG. However, our study reported lower sensitivity rates compared to those reported in a recent systematic review and meta‐analysis [20]. This discrepancy may stem from differences in study populations. While some studies focused on moderate‐to‐severe AG or included the full spectrum of gastric atrophy, our cohort had a relatively high proportion of mild‐to‐moderate AG, potentially presenting greater detection challenges for both LFA and ELISA. Additionally, variability in interobserver agreement during histological evaluations could contribute to differences in diagnostic performance across studies.

Although LFA and ELISA demonstrated similar performance in detecting AG, a weaker correlation was observed between the two assays in *H. pylori* IgG titers. Nevertheless, both methods demonstrated similar sensitivity and specificity when histological findings served as the reference standard. This apparent discrepancy can be explained by the fact that correlation reflects agreement in continuous IgG titer values, whereas sensitivity and specificity are measured based on binary classification against the reference standard. Therefore, even with the limited correlation, the comparable sensitivity and specificity suggest that both assays are similarly effective in identifying histologically confirmed infections when applying their respective thresholds. The weaker correlation may be attributed to differences in antigen composition between the two assays. LFA employs a single *H. pylori* antigen, while ELISA uses a cocktail of multiple antigens. This distinction may influence binding affinity and detection outcomes. Furthermore, geographic variations in *H. pylori* strains could further complicate cross‐assay comparisons [21]. For instance, selecting antigens from *cagA*‐positive *H. pylori* strains, more prevalent in the East Asian, may enhance diagnostic performance in certain populations but reduce cross‐reactivity in others. The ethnically diverse population in the Rotterdam region of the Netherlands may have also contributed to the observed variability in *H. pylori* detection between the assays. These findings underscore the need for further validation of *H. pylori* detection to ensure more consistency and reliability in point‐of‐care applications.

Our study categorized corpus AG into AIG and non‐AIG based on the presence of anti‐parietal cell antibodies. Consistent with our previous findings, the combination of a reduced PGI/PGII ratio and elevated G‐17 effectively differentiated AIG [18]. Similarly, other studies have shown that patients with AIG often exhibit more severe corpus destruction, leading to significantly lower PGI levels, which could help identify AIG [22]. These findings provide insights into the identification of AIG in corpus AG and could facilitate proactive targeted testing for AIG, such as APCA detection. This approach may enable early detection of AIG during the screening programs, helping prevent disease progression. Although the role of AIG in gastric cancer development remains disputed [23], MAPS III Guideline currently recommends endoscopic surveillance every 3 years for patients with AIG to monitor disease progression [6].

A key strength of this study is the direct head‐to‐head comparison of LFA and ELISA within a well‐defined cohort, enabling a robust assessment of their diagnostic performance in detecting AG. While both methods demonstrate comparable accuracy, LFA offers distinct advantages in clinical practice, particularly in terms of turnaround time and cost‐effectiveness. ELISA typically requires dedicated laboratory infrastructure, skilled personnel, and multiple incubation and washing steps, all of which contribute to longer processing times and higher operational costs. In contrast, LFA could be performed rapidly at the point of care with minimal technical expertise and equipment. This enables results to be obtained within minutes and significantly reduces the overall cost per test. The simplicity, affordability, and rapid results of LFA not only streamline the diagnostic process but also support timely clinical decision‐making and broader accessibility, particularly in resource‐limited settings. These attributes enhance the utility of LFA for early AG detection, potentially preventing the progression to more severe conditions such as gastric cancer. Furthermore, our study confirmed the utility of a reduced PGI/PGII ratio and the elevated G‐17 levels as valuable biomarkers for AIG detection.

Nevertheless, we also acknowledge several limitations in our study. First, only 79 out of 157 patients with gastric atrophy had OLGIM stage II–IV. The interobserver and intraobserver variability in assessing mild mucosal changes could influence AG diagnosis when histology is used as the reference standard. Second, histological evaluation served as the reference standard for *H. pylori* detection, which is inherently limited by the patchy distribution of *H. pylori* in gastric mucosa, potentially leading to sampling errors [24]. Furthermore, *H. pylori* density often declines significantly in AG, increasing the risk of false‐negative results [25]. Lastly, this study utilized fasting serum samples for LFA evaluation, although this assay could deliver faster results using fingerprick blood samples. The use of different sample types may influence diagnostic performance, as capillary blood obtained via fingerprick represents a mixture of arterial, venous, and interstitial fluids, and may differ in analyte concentrations compared to venous serum. Therefore, further studies are needed to validate the broader clinical utility of LFA in AG detection across diverse settings.

In conclusion, our study demonstrates that LFA provides diagnostic performance comparable to ELISA for detecting AG, making it a promising screening tool. However, due to its limited sensitivity and high specificity, a negative LFA result cannot definitively exclude AG. Therefore, LFA may serve as a valuable supplementary tool, particularly in point‐of‐care settings, to enhance screening programs and improve early detection of AG.

## Author Contributions

**Luochengling Xiang, Xiaopei Guo, Maikel P. Peppelenbosch, Manon C. W. Spaander, Gwenny M. Fuhler:** study concept and design. **Luochengling Xiang, Ying Zhou, Xiaopei Guo, Michiel C. Mommersteeg, Stella A. V. Nieuwenburg:** data acquisition. **Luochengling Xiang:** conduct of ELISA and LFA assays. **Luochengling Xiang, Ying Zhou:** statistical analysis. **Luochengling Xiang, Gwenny M. Fuhler:** manuscript drafting. **Maikel P. Peppelenbosch, Manon C. W. Spaander, Gwenny M. Fuhler:** study supervision. All authors: critical revision of the manuscript for important intellectual content.

## Conflicts of Interest

The authors declare no conflicts of interest.

## Supporting information


**Figure S1:** hel70066‐sup‐0001‐FigureS1.docx.

## Data Availability

The data that support the findings of this study are available from the corresponding author upon reasonable request.
