# Supplementary material for: Evaluation of Pepsinogen I, II, Gastrin 17 and Helicobacter pylori IgG in Atrophic Gastritis: A Head‐To‐Head Comparison of Lateral Flow and Enzyme‐Linked Immunosorbent Assays
Source: Helicobacter. 2025 Aug 19;30(4):e70066. doi: 10.1111/hel.70066 (PMC12365252; doi:10.1111/hel.70066)
Supplement: Supplementary file 1 — Figure S1: hel70066‐sup‐0001‐FigureS1.docx. [file HEL-30-e70066-s001.docx]

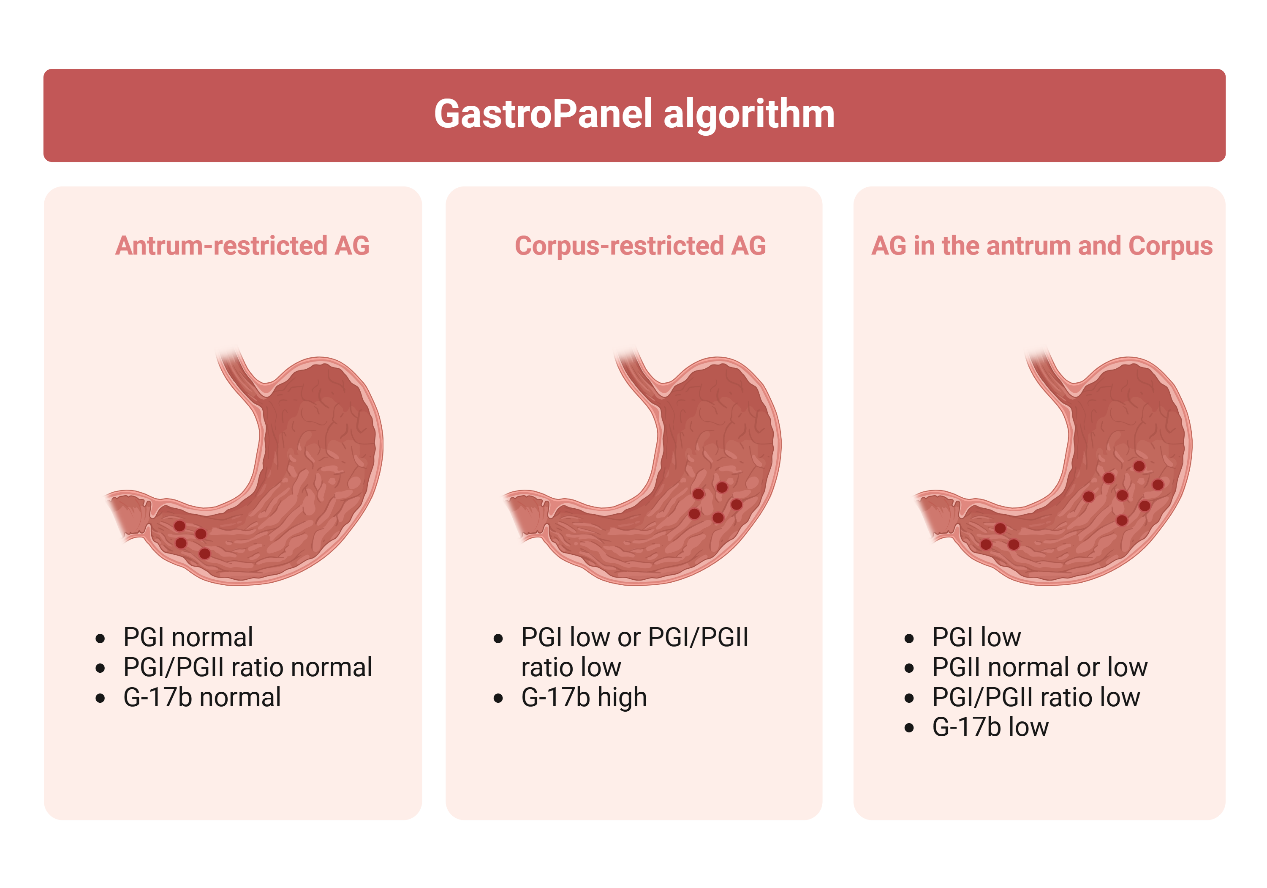


**Figure S1** GastroPanel algorithm for classifying atrophic gastritis (AG). GastroPanel algorithm was applied to determine the location of atrophic gastritis, based on pepsinogen I (PGI), pepsinogen II (PGII), PGI/PGII ratio, gastrin-17 (G-17) measured by LFA and ELISA. Overall AG was classified into antrum-restricted AG, corpus-restricted AG and AG in the antrum and corpus.
